# Chronic Pruritus Responding to Dupilumab—A Case Series

**DOI:** 10.3390/medicines6030072

**Published:** 2019-06-29

**Authors:** Lisa L. Zhai, Kevin T. Savage, Connie C. Qiu, Annie Jin, Rodrigo Valdes-Rodriguez, Nicholas K. Mollanazar

**Affiliations:** 1 Department of Dermatology, Lewis Katz School of Medicine at Temple University, Philadelphia, PA 19140, USA; 2 Lewis Katz School of Medicine, Temple University, Philadelphia, PA 19140, USA; 3 Drexel University College of Medicine, Philadelphia, PA 19129, USA; 4 Department of Dermatology, University of Virginia, Charlottesville, VA 22903, USA; 5 Department of Dermatology, Perelman School of Medicine, University of Pennsylvania, Philadelphia, PA 19140, USA

**Keywords:** dupilumab, IL-4, IL-13, pruritus, chronic pruritus of unknown origin, prurigo nodularis, uremic pruritus, lichen planus, eosinophilic dermatosis of hematologic malignancy, chronic pruritus

## Abstract

**Background:** Chronic pruritus is defined as itch lasting for greater than six weeks. Pruritus is a burdensome manifestation of several internal and external disease states with a significant impact on quality of life. Dupilumab has shown promise in treating a number of conditions including atopic dermatitis (AD) and asthma. Its success in reducing pruritus in AD has generated interest regarding its potential application in other pruritic conditions, such as chronic pruritus of unknown origin, uremic pruritus, and pruigo nodularis. **Methods:** In this retrospective analysis, we present a series of 20 recalcitrant pruritus patients seen at a tertiary center treated with off-label dupilumab at standard AD dosing. **Results:** Dupilumab was successful at reducing itch in all treated patients, leading to complete resolution in 12/20 patients and an overall mean NRSi reduction of 7.55. Dupilumab was well tolerated with no significant adverse effects. **Conclusions:** Our case series suggests dupilumab may be a safe and efficacious therapeutic option in several pruritic conditions and demonstrates the need for further studies to better ascertain its place in the pruritus treatment armamentarium.

## 1. Introduction

Pruritus, the Latin word for itch, is defined as an “unpleasant sensation that elicits the desire or reflex to scratch” [1]. Perhaps unsurprisingly, pruritus is an extremely common complaint. Over an 11-year period in America alone, pruritus accounted for 1% of all physician visits, bringing the 11-year total to a staggering 77 million visits [2]. In comparison, 1.8% of all physician visits in America were for low back pain [2]. Undoubtedly, these numbers underestimate the extent of the true problem, as studies have demonstrated that only half of patients experiencing pruritus will visit a physician for that problem [2,3,4,5,6]. 

Pruritus is separated into two clinical categories: acute and chronic. Chronic pruritus (CP) is defined as itch lasting more than six weeks, while acute pruritus (AP) is defined as itch lasting less than six weeks [3]. As many clinicians can attest, antihistaminergic treatments fail to provide patients suffering from CP with any meaningful benefits [7]. This anecdotal finding leads to the hypothesis that there must be an additional pathway besides just histaminergic itch. Indeed, pruritus can further be divided into histaminergic and non-histaminergic itch. We now know that the neurophysiologic and neuroanatomical pathways for histaminergic and non-histaminergic itch, while related, are entirely separate and independent from one another [8,9,10]. The psychosocial burden of CP and its significant negative impact on quality of life are now well understood. Recently, it was reported that the effects of CP are as debilitating as chronic pain [11,12,13,14]. Despite all these recent advances in our understanding of CP, the underlying pathophysiologic mechanisms of CP are not fully elucidated and currently, there are no medications that are specifically FDA approved for the treatment of this debilitating disease. Since CP is associated with myriad systemic and primary dermatologic diseases, there likely is not one single cause of CP. Rather, CP is likely caused by a complex interface between skin, keratinocytes, cutaneous nerve fibers, cytokines, pruritogens, and the peripheral and central nervous systems [15]. 

While there may not be one single cause of CP, the final neuronal pathway that carries the itch signal from the periphery to the central nervous system may be a conserved constant. Recent bench-to-bedside work has implicated the cytokine IL-4 in the neuronal processes that drive CP. In their seminal publication, Oetjen et al., demonstrated: the receptor for IL-4, IL-4Ra, is directly expressed on sensory neurons in both mice and human dorsal root ganglia; that expression of Th2 cytokines (IL-4, IL-13, and IL-31) directly activates sensory dorsal root ganglia neurons; and that ablation of IL-4Ra abated chronic itching in a murine mouse model [16,17]. Taken together, these findings demonstrated that CP, at least in part, is dependent on neuronal IL-4RA signaling. With the aforementioned findings in mind, it was hypothesized that targeting IL-4RA signaling might act to ablate the itch sensation, regardless of the underlying cause of CP. 

In 2018, we reported significant reductions in CP in patients with prurigo nodularis using dupilumab, a novel monoclonal IL-4/IL-13 antagonist approved for the treatment of moderate to severe atopic dermatitis (AD) [18]. Dupilumab is a fully human monoclonal IgG antibody that occupies the shared alpha subunit receptor site for IL-4, which blocks the effects of the IL-4 and IL-13 signaling pathway [15]. IL-4 and IL-13 are thought to be key mediators in the chronic pruritus that is the hallmark of (AD), and are key upstream drivers in the Th2 pathways that modulate myriad downstream targets, such as IL-5 and IL-31 [8,19]. Herein, we present a case-series of 20 patients with CP, of various causes, treated off-label in a busy tertiary academic referral center with dupilumab. 

## 2. Report of Cases

We retrospectively reviewed 20 patients that presented to our tertiary referral center with pruritic skin conditions including chronic pruritus of unknown origin (CPUO), eosinophilic dermatosis of hematologic malignancy (EDHM), lichen planus, prurigo nodularis (PN), and uremic pruritus. Only one patient had a known history of AD as a child. All patients failed topical steroids and topical calcineurin inhibitors. Subcutaneous injections of dupilumab (Dupixent; Regeneron-Sanofi) were administered in the standard AD dosing regimen (600 mg induction dose followed by 300 mg every 2 weeks thereafter). Baseline patient-reported numeric rating scale itch intensity (NRSi) was recorded for each patient prior to therapy initiation. At each subsequent visit, patients reported their NRSi. Mean NRSi reduction was calculated for each subject using their baseline NRSi and the NRSi from their last visit while taking dupilumab. Total itch reduction was defined as an NRSi of 0.

### 2.1. All Patients with Chronic Pruritus (n = 20)

Mean NRSi (SD): 9.00 (1.21)

Mean NRSi Reduction (SD): 7.55 (2.68)

**Figure 1:**
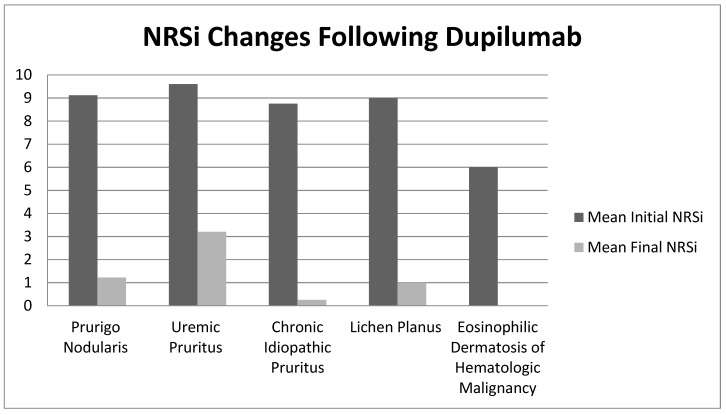
Numeric rating scale itch intensity (NRSi) changes following dupilumab.

**Figure 2:**
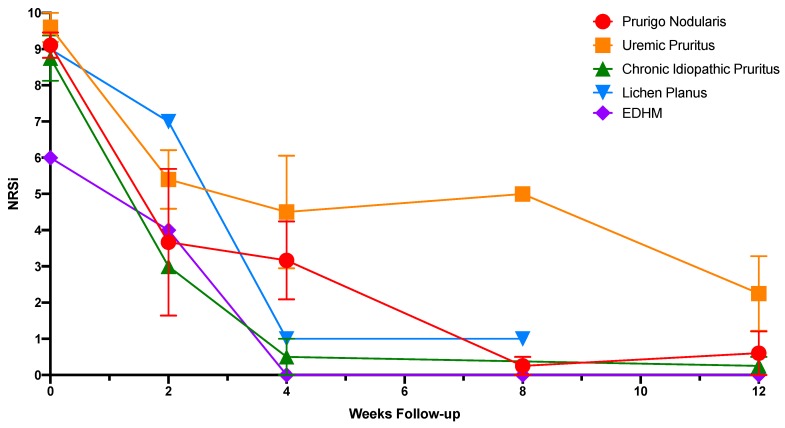
Response to dupilumab by disease over time.

In total, 20 patients were treated with dupilumab for chronic pruritus (all causes), with between one and five follow up visits (Table 1). Of the 20 patients, 15 had follow-up data at 12 weeks or longer. This cohort demonstrated an initial mean NRSi of 9.00 (SD) (1.21) and a mean NRSi reduction (SD) of 7.55 (2.68) (Table 1 and Figure 1). The trend of response to dupilumab over time can be seen in Figure 2. There was no statistically significant difference with regards to response based on gender. Meaningful statistical significance could not be determined in regard to the rapidity of response due to the small sample size and variance in follow-up time intervals.

### 2.2. Prurigo Nodularis (n = 9)

Mean Baseline NRSi (SD): 9.11 (1.05)

Mean NRSi Reduction (SD): 7.89 (2.93)

Nine patients with PN had an initial mean NRSi (SD) of 9.11 (1.05) (Table 2), with between one to five follow-up visits. Of the nine patients, six had follow-up data at 20 weeks or longer. This cohort demonstrated a mean NRSi reduction (SD) of 7.89 (2.93). One patient (denoted with * in Table 2) responded so well that at 8-week follow-up, they elected to go off treatment and remained itch free at 20-week follow-up off treatment.

### 2.3. Uremic Pruritis (n = 5)

Mean Baseline NRSi (SD): 9.60 (0.89)

Mean NRSi Reduction (SD): 6.40 (3.51)

Five patients with uremic pruritus had an initial mean NRSi (SD) of 9.60 (0.89) (Table 3), with between three to five follow-up visits. Of the five patients, four had follow-up data at 12 weeks. This cohort demonstrated a mean NRSi reduction (SD) of 6.40 (3.51). One patient was hemodialysis dependent while the other patients were not. 

### 2.4. Chronic Idiopathic Pruritis (n = 4)

Mean Baseline NRSi (SD): 8.75 (1.26)

Mean NRSi Reduction (SD): 8.5 (1.29)

Four patients with CPUO had an initial mean NRSi (SD) of 8.75 (1.26) (Table 4) All four patients had follow-up data at 12 weeks or greater. This cohort demonstrated a mean NRSi reduction (SD) of 8.50 (1.29) (Table 4). Two patients (denoted by * in Table 4) responded so well that they elected to go off treatment at their 4-week follow-up and remained itch free at 20-week follow-up or greater. 

### 2.5. Lichen Planus (n = 1)

Mean Baseline NRSi: 9.0

Mean NRSi Reduction: 8.0

One patient with LP reported an initial mean NRSi of 9, with 3 follow up visits spanning 8 weeks (Table 1). The patient reported a mean NRSi reduction of 8. The patient had previously failed trials of prednisone and acitretin, as well as topical steroids and topical calcineurin inhibitors. Of note, both the patient’s rash and itch were noted to improve substantially on treatment. 

### 2.6. Eosinophilic Dermatosis of Hematologic Malignancy (n = 1)

Mean Baseline NRSi: 6.0

Mean NRSi Reduction: 6.0

One patient with EDHM reported an initial mean NRSi of 6, with 4 follow-up visits spanning 16 weeks (Table 1). Prior to initiation of dupilumab, the patient had more severe pruritus that responded to systemic steroids. After 3 courses of month-long prednisone tapers in as many months, the decision was made to switch the patient to a steroid sparing agent. The patient’s hematologist was not comfortable with immunosuppressive medications, such as methotrexate, as the patient was receiving chemotherapy for CLL. In the data reported, the patient used only dupilumab and topical corticosteroids as needed. 

## 3. Discussion

Pruritus is mediated through both histaminergic and non-histaminergic pathways [5]. Histaminergic itch is induced by the histamine pathway while non-histaminergic itch appears to be mediated by proteinase activated receptors [8,15]. Cutaneous sensory nerves originate from dorsal root ganglion and terminate in the dermis or epidermis close in proximity to skin cells such as keratinocytes, Langerhans cells, and fibroblasts [15]. Both histaminergic and non-histaminergic itch signals are relayed to the brain via the spinal thalamic tract (STT), but the two do not converge on the same STT neurons [8]. Brain functional imaging studies show that different brain regions, in addition to a core group of brain structures, are selectively activated depending on the type of itch induced [8]. Thus, the distinction between histaminergic and nonhistaminergic itch is seen throughout transmission of the itch signal from the distal periphery to the cortex. This distinction between the two itch pathways explains the clinical quagmire that has vexed physicians for the past several decades regarding the treatment of non-histaminergic itch—traditional anti-histaminergic mediated therapies simply do not work in these patients. All treatments used by clinicians, including commonly used modalities such as phototherapy, are entirely off-label, based on limited case reports, and often carry significant risks. The recent successful report of a Phase 2 clinical trial for Serlopitant (Menlo Therapeutics, Redwood City, CA), a selective NK_1_R antagonist, in the treatment of CP is promising and a step in the right direction [17,20]. Nevertheless, there remains an unmet need regarding additional therapeutic agents for the treatment of CP in clinical practice.

We present quantitative data regarding itch intensities and respective reductions from baseline in 20 patients with chronic pruritus. Our mean NRSi of 9.00 is substantially higher when compared to previously reported itch intensities [20]. Most likely, our reported mean itch intensity is significantly higher than previous reports as this cohort represents only patients with severe and substantial disease, which necessitated off-label use of a systemic medication. We report an overall mean NRSi reduction (SD) of 7.55 (2.68), which is on the higher end of previous reports [20]. The higher degree of response reported by our patients in this case series may indicate that dupilumab is effective at abolishing the itch sensation, regardless of the underlying cause. The response to dupilumab in our cohort may be explained by recent bench-to-bedside work, wherein IL-4Ra was found to be expressed directly on sensory neurons in both mice and human dorsal root ganglia [16]. Blockade of the IL-4ra signaling with dupilumab may block neuronal transmission of the itch signal from the periphery to the CNS. 

### 3.1. Prurigo Nodularis 

PN was the single largest etiology of CPUO in our cohort (n = 9). The exact prevalence of PN is unknown, though PN is more prevalent in the elderly and in patients with atopy [21]. PN is associated with numerous comorbidities including HIV, cardiovascular disease, and psychiatric illness [22]. Itch from PN is particularly bothersome; in a tertiary itch center, PN patients experienced the worst initial itch intensities and responded the least to treatment [17]. Our patients reported a mean NRSi of 9.11, which is in line from previous reported itch intensities in PN [20]. 

Although PN pathogenesis is incompletely understood, tryptase, IL-31, prostaglandins, and neuropeptides have all been implicated [23,24]. IL-31 mRNA is markedly elevated in lesional dermis compared to healthy skin [25]. PN patients demonstrate characteristic dermal hypersensitivity resultant from neuronal hyperplasia in the dermis [26]. In the epidermis, however, the opposite is true as there is neuronal hypoplasia, and healed prurigo nodules may demonstrate increased nerve fiber density [27]. Successful treatment of PN with dupilumab has been reported in the literature [18,28,29]. There is evidence to suggest that PN is a Th2 cytokine dependent process, as epidermal biopsies of lesional skin in PN patients show higher levels of STAT6 compared to controls [30]. Importantly, STAT6 is activated in part by IL-4/IL-13 [30]. 

Neuropeptides also appear to play a role in PN development as PN patients demonstrate an increased number of substance P (SP)-dependent nerve fibers in the skin [31]. SP is a neuropeptide released by activated sensory neurons in the skin and appears to be an important modulator for non-histaminergic itch [32,33]. Substance P binds neurokinin 1 receptor (NK_1_R) present on mast cells leading to release of pro-pruritic mediators [15]. NK_1_R is involved in modulating SP signaling. Serlopitant has shown promise in PN treatment [34,35]. In a randomized clinical trial of serlopitant vs placebo for PN treatment, serlopitant, a selective NK_1_R antagonist, provided a significantly better reduction in NSRi versus placebo through eight weeks of treatment [35]. However, treatment-emergent adverse events were experienced by nearly 75% of patients in the treatment arm, suggesting that serlopitant therapy is not without its drawbacks. Additionally, pregabalin, a neuroleptic, has shown promise in treating PN, illustrating statistically significant decreases in mean itch VAS scores among 30 patients [35,36,37]. 

Dupilumab was highly effective at relieving itch in the nine PN patients presented in this series. Despite demonstrating a high mean initial NRSi (9.11), all saw a reduction in itch, with most completely resolving (n = 7) and a mean NRSi reduction (SD) of 7.89. These results are encouraging, as patients treated with more traditional therapies including betamethasone [38] (mean NRSi reduction: 4.9) and pimecrolimus [39] (mean NRSi reduction: 2.7) saw markedly lower mean reductions. More potent immunotherapies including cyclosporine and thalidomide may be efficacious; however, they carry significant risks [40,41,42]. Our mean NRSi reductions are higher than previous reports from a tertiary itch center, but slightly lower compared to more recent reports on dupilumab in the treatment of PN (mean NRSi reduction: 8.8). Our slightly lower reduction might be due to the fact that we have over double the number of patients previously reported. The rapid and profound response noted in our patients further implicates IL-4/IL-13 in the pathophysiology of PN. Of note, no adverse events were experienced by our nine patients, a striking difference when compared to serlopitant. Randomized controlled trials in PN using dupilumab are needed. 

### 3.2. Chronic Pruritus of Unknown Origin

Chronic pruritus of unknown origin (CPUO) is a devastating and burdensome pruritic condition. CPUO disproportionally affects the elderly, consistent with data from our cohort (n = 4, average age = 63 years) [43]. An underlying immune dysregulation is suspected in CPUO development and maintenance; a study of four elderly patients with CPUO showed marked eosinophilia and IgE levels in biopsies of affected skin [44], consistent with a skewed Th2 immune response. IL-4’s interaction with lymphocytes and resident myelocytes—in conjunction with IL-13—are important drivers of Th2 mediated disease states [45]. Additionally, age-related loss of protective Th1 immune cells may hasten the Th2 immune response that is characteristic of this condition [46]. CPUO treatment has traditionally revolved around antihistamine and more recently, anti-IgE therapies, with limited effect [47]. Dupilmab, however, has shown efficacy in treating a wide array of Th2 dependent diseases including AD and asthma, likely through modulation of IL-4 and IL-13’s interaction with immune cells [48,49,50]. 

The mean initial NRSi of our cohort with CPUO was 8.75, which is not dissimilar from previous reports from a tertiary itch center (NRSi: 8.2). Dupilumab effectively treated itch in all our patients suffering from CPUO, with a mean NRSi reduction (SD) of 8.50 (1.29), which is significantly greater when compared to previous reports showing a mean NRSi reduction of 2.8 [17]. No adverse effects were reported in this cohort. 

Dupilumab offered encouraging results in treating CPUO, but it is far from the only treatment used in managing this condition. Naltrexone, a partial antagonist of µ, κ, and δ opioid receptors has demonstrated efficacy in CP treatment as well [51]. Naltrexone exerts its anti-pruritic effect by directly binding opioid receptors in the skin, blocking mast cell, basophil, and IgE-mediated histamine release [52]. Among patients with pruritus caused by underlying systemic disease, 50 mg naltrexone daily resulted in a significant therapeutic response in 70% of patients in just one week, suggesting rapid onset of efficacy [53]. A systematic review of naltrexone use in chronic inflammatory dermatologic conditions found that both high and low dose naltrexone may be efficacious in treating pruritic disease [52]. Naltrexone is a promising new therapy in the treatment of CP. Both naltrexone and dupilumab warrant further controlled trials in the treatment of CPUO.

### 3.3. Lichen Planus

Lichen Planus (LP) is a cell-mediated immune response of unknown origin that may affect the skin, oral cavity, nails, scalp, genitalia, or esophagus. LP classically presents with pruritic, polygonal, violaceous, flat-topped papules and plaques [54]. LP may be self-limiting and resolve spontaneously within two years. Topical corticosteroids are first-line therapy for all forms of LP, including cutaneous, genital, and mucosal erosive lesions. Systemic therapy with acitretin or an oral immunosuppressant should be considered for patients with severe LP that does not respond to topical treatment [55]. Pruritus is a common complaint by patients with LP, with one study of 30 patients reporting a prevalence of 96.7% [56]. Pruritus is an important and burdensome symptom of LP that is largely unstudied. Pathogenesis of itch in LP has not been fully elucidated, and there are no effective therapeutic modalities alleviating pruritus in patients suffering from this disease [57]. What little literature does exist on the pathophysiology of LP is largely focused on oral manifestations of the disease. When compared to healthy controls, patients with LP have elevated levels of IL-6 in their serum [58]. Furthermore, IL-6 levels correlate with LP disease severity and have been suggested as a possible surrogate marker for disease activity [59,60]. IL-6 is implicated in promoting IL-4 induced Th2 processes and in inhibition of IL-12 induced Th1 pathway [61]. 

Our LP patient presented with a chronic and severe form of the condition. The patient reported that the associated pruritus caused substantial distress. On dupilumab, our patient experienced improvement in rash and NRSi within one month. The rapidity of improvement and self-reported patient satisfaction indicates that dupilumab may begin to address the unmet need for anti-pruritics in LP.

### 3.4. Uremic Pruritus

Uremic pruritus (UP), or chronic kidney disease associated pruritus (CKD-aP), is a distressing and frequent symptom in chronic renal failure. UP has been associated with poorer quality of life, and depression [62]. A large, international study estimated the prevalence of moderate to extreme pruritus among patients with end-stage kidney disease on hemodialysis to be 42% [63]. The pathogenesis of UP is not well elucidated, but studies have implicated interleukin-31 (IL-31), which is upregulated by Th2 cells in pruritic disorders such as AD and cutaneous T-cell lymphoma [64]. A systemic review of treatments for UP revealed that with the exception of evidence for gabapentin, there remains considerable uncertainty regarding the efficacy of other treatments for UP [65]. Gabapentin is an analog of γ-aminobutyric acid, though it does not interact with γ-aminobutyric acid receptors [66,67]. Gabapentin may modulate itch in the dorsal root ganglion and dorsal horn of the spinal cord by inhibiting the α2δ subunit of voltage-dependent calcium channels, thereby increasing the threshold for neuronal excitation [36,67,68]. Gabapentin significantly reduced itch in hemodialysis patients suffering from UP in a randomized clinical trial, decreasing pruritus scores on a VAS from 8.4 at baseline to 1.2 following treatment. Side effects were limited to dizziness and fatigue, suggesting that gabapentin may be an efficacious anti-pruritic therapy [69,70]. Phototherapy, neuroleptics, antidepressants, and many other treatment modalities are used off-label for UP, each with variable short-term efficacy, limited long-term efficacy, and potential for numerous serious adverse events [71].

Our UP patients all reported improvement in NRSi. Of note, response to dupilumab was slower, and the rate of recurrence was higher than what was observed with other patients in this series. The reasons for this phenomenon are unknown. Perhaps the efficacy of dupilumab is dampened by dialysis, although this would not fully explain the discrepancy in this cohort, as only one patient was HD dependent. Our findings demonstrate the need for further studies regarding the pathophysiology of UP—as treatments may elude us until there is a better understanding of the disease mechanisms. 

### 3.5. Eosinophilic Dermatosis of Hematologic Malignancy (EDHM) 

EDHM is a unique eosinophilic skin eruption described in patients with hematologic malignancies that was previously called exaggerated arthropod bite reaction, terminology used in part because the lesions resemble arthropod bites both clinically and histologically [72]. In 2001, Bryd et al. coined the term eosinophilic dermatosis of myeloproliferative disease to refer to eosinophilic eruptions in patients with hematologic disorders [73]. Davis et al noticed a striking resemblance of these lesions to the previously described exaggerated arthropod bites [74]. Notably, their previous study revealed that only 25% of their patients with lesions resembling arthropod bites with prominent eosinophilia had a history of arthropod bites [75]. Accordingly, the lesions were determined to be of the same entity and the term eosinophilic dermatosis of myeloproliferative disease was deemed more accurate [73,74]. As more cases were reported, the term eosinophilic dermatosis of hematologic malignancies became preferred to better encompass the variety of hematologic malignancies associated with the eruption [76,77,78].

Our EDHM patient presented with an ongoing history of chronic lymphocytic leukemia (CLL), the most commonly associated hematologic malignancy reported with EDHM, although EDHM has been described in the context of acute lymphoblastic leukemia, acute monocytic leukemia, large cell lymphoma, mantle cell lymphoma, and myelofibrosis [76,79]. Previous studies reported that most cases do not respond sufficiently to topical corticosteroids, systemic antihistamines, UV-B phototherapy, or interferon therapy [75]. Several reports have documented partial or complete response to systemic prednisone, which was true for our patient as well [72,80]. Previous studies have also found dapsone to be successful initially, but patients on dapsone had to be discontinued due to side effects [80]. 

The pathogenesis of EDHM is thought to be due to an imbalance of IL-4 and IL-5, an excess of which is hypothesized to lead to the proliferation of neoplastic B cells considered to be the primary driver of the eruption; importantly, IL-4 is well known to induce B-cell class switching [73,76,78,81,82]. In theory, the mechanism of dupilumab should normalize the excess IL-4, mitigating an instigating factor in the development of the lesions. On dupilumab, our patient experienced a rapid clearing of the rash and a steep improvement of NRSi within two months. The complete and sustained response with the lack of side effects, highlights dupilumab as a possible treatment to fill the unmet need of successful therapeutics in EDHM. 

## 4. Conclusions

Our findings re-capitulate previous reports regarding the success of dupilumab in the treatment of prurigo nodularis and further elaborate on its ability to alleviate pruritus in numerous other itchy dermatoses. Specifically, dupilumab resulted in significant improvement of CP in all 20 patients presented in this series. The magnitude and rapidity of improvement, as well as the absence of significant adverse events further support the need to explore dupilumab as a therapy for CP, ideally in a randomized, controlled fashion. 

## Figures and Tables

**Table 1:** Summary data.

Patient	A	B	C	D	E	F	G	H	I	J	K	L	M	N	O	P	Q	R	S	T
**Age**	72	65	66	56	62	82	65	57	52	28	71	78	43	64	55	58	55	63	49	67
**Sex**	F	M	F	F	M	M	F	F	M	F	F	M	F	M	F	F	F	M	M	M
**PMHx Atopic Derm**	No	No	No	Yes	No	No	No	No	No	No	No	No	No	No	No	No	No	No	No	No
**Diagnosis**	Prurigo nodularis	Chronic pruritus of unknown origin	Chronic pruritus of unknown origin	Chronic pruritus of unknown origin	Uremic pruritis	Eosinophilic dermatosis of hematologic malignancy	Chronic pruritus of unknown origin	Uremic pruritis	Lichen planus	Prurigo nodularis	Uremic pruritis	Uremic pruritis	Prurigo nodularis	Uremic Pruritus	Prurigo nodularis	Prurigo nodularis	Prurigo nodularis	Prurigo nodularis	Prurigo nodularis	Prurigo nodularis
**Initial NRSi**	9/10	9/10	7/10	10/10	8/10	6/10	9/10	10/10	9/10	9/10	10/10	10/10	10/10	10/10	8/10	10/10	10/10	7/10	9/10	10/10
2-week follow-up NRSi	-	-	3/10	-	7/10	4/10	3/10	6/10	7/10	7/10	4/10	7/10	-	3/10	-	-	-	4/10	0/10	-
4-week follow-up NRSi	-	-	0/10*	1/10*	7/10	0/10	-	6/10	1/10	6/10	-	5/10	0/10	0/10	5/10	-	3/10	0/10	-	5/10
8-week follow-up NRSi	0/10*	-	-	-	-	0/10	2/10	5/10	1/10	-	-	5/10	0/10	-	-	-	1/10	-	-	0/10
12-week follow-up NRSi	0/10	-	-	-	-	0/10	1/10	4/10	-	-	1/10	4/10	-	0/10	-	3/10	0/10	0/10	-	0/10
16-week follow-up NRSi	-	-	-	0/10	-	-	-	-	-	-	-	-	-	-	-	-	-	-	-	-
20-week follow-up NRSi	0/10	-	-	-	-	-	-	-	-	-	-	-	0/10	0/10	-	0/10	0/10	0/10	-	0/10
24-week follow-up NRSi	-	1/10	-	0/10	-	-	-	-	-	-	-	-	-	-	-	0/10	-	-	-	-
28-week follow-up NRSi	-	-	0/10	-	-	-	-	-	-	-	-	-	-	-	-	-	-	-	-	-
32-week follow-up NRSi	-	-	-	-	-	-	-	-	-	-	-	-	-	-	-	-	-	-	-	-
36-week follow-up NRSi	-	-	-	-	-	-	-	-	-	-	-	-	-	-	-	0/10	0/10	-	-	-
40-week follow-up NRSi	-	-	-	-	-	-	-	-	-	-	-	-	-	-	-	-	-	-	-	-
44-week follow-up NRSi	-	-	-	-	-	-	-	-	-	-	-	-	-	-	-	-	-	-	-	-
48-week follow-up NRSi	-	-	-	-	-	-	-	-	-	-	-	-	-	-	-	-	-	-	-	-
52-week follow-up NRSi	-	-	-	-	-	-	-	-	-	-	-	-	-	-	-	-	-	-	-	-
> 1 year follow-up NRSi	-	0/10	-	-	-	-	-	-	-	-	-	-	-	-	-	0/10	-	-	-	-
NRSi Reduction	9	9	7	10	1	6	8	6	8	3	9	6	10	10	3	10	10	7	9	10
**Total Patients**	**20**																			
**Mean Initial NRSi (SD)**	**9 (1.21)**																			
**Mean NRSi Reduction (SD)**	**7.55 (2.68)**																			

* Discontinued treatment following this visit due to continued pruritus relief.

**Table 2:** Prurigo nodularis.

Patient	A	J	M	O	P	Q	R	S	T	
**Age**	72	28	43	55	58	55	63	49	67	
**PMHx Atopic Derm**	No	No	No	No	No	No	No	No	No	
**Diagnosis**	Prurigo nodularis	Prurigo nodularis	Prurigo nodularis	Prurigo nodularis	Prurigo nodularis	Prurigo nodularis	Prurigo nodularis	Prurigo nodularis	Prurigo nodularis	
**Initial NRSi**	9/10	9/10	10/10	8/10	10/10	10/10	7/10	9/10	10/10	
2-week follow-up NRSi	-	7/10	-	-	-	-	4/10	0/10	-	
4-week follow-up NRSi	-	6/10	0/10	5/10	-	3/10	0/10	-	5/10	
8-week follow-up NRSi	0/10	-	0/10	-	-	1/10	-	-	0/10	
12-week follow-up NRSi	0/10*	-	-	-	3/10	0/10	0/10	-	0/10	
16-week follow-up NRSi	-	-	-	-	-	-	-	-	-	
20-week follow-up NRSi	0/10	-	0/10	-	0/10	0/10	0/10	-	0/10	
>28 week follow-up NRSi	-	-		-	0/10	0/10		-		
NRSi Reduction	9	3	10	8	10	10	7	9	10	
**Total patients**	**9**									
**Mean Initial NRSi (SD)**	**9.11 (1.05)**									
**Mean NRSi Reduction (SD)**	**7.89 (2.93)**									

* Discontinued treatment after 8 week follow-up visit due to continued pruritus relief.

**Table 3:** Uremic pruritus.

Patient	E	H	K	L	N
**Age**	62	57	71	78	64
**PMHx Atopic Derm**	No	No	No	No	No
**Diagnosis**	Uremic pruritis	Uremic pruritis	Uremic pruritis	Uremic pruritis	Uremic Pruritus
**Initial NRSi**	8/10	10/10	10/10	10/10	10/10
**2-week follow-up NRSi**	7/10	6/10	4/10	7/10	3/10
**4-week follow-up NRSi**	7/10	6/10	-	5/10	0/10
**8-week follow-up NRSi**	-	5/10	-	5/10	-
**12-week follow-up NRSi**	-	4/10	1/10	4/10	0/10
**NRSi Reduction**	1	6	9	6	10
**Total patients**	**5**				
**Mean Initial NRSi (SD)**	**9.6 (0.89)**				
**Mean NRSi Reduction (SD)**	**6.4 (3.51)**				

**Table 4:** Chronic pruritus of unknown origin.

Patient	B	C	D	G
**Age**	65	66	56	65
**PMHx Atopic Derm**	No	No	Yes	No
**Diagnosis**	Chronic pruritus of unknown origin	Chronic pruritus of unknown origin	Chronic pruritus of unknown origin	Chronic pruritus of unknown origin
**Initial NRSi**	9/10	7/10	10/10	9/10
**2-week follow-up NRSi**	-	3/10		3/10
**4-week follow-up NRSi**	-	0/10*	1/10*	-
**12-week or > follow-up NRSi**	0/10	0/10	0/10	1/10
**NRSi Reduction**	9	7	10	8
**Total patients**	**4**			
**Mean Initial NRSi (SD)**	**8.75 (1.26)**			
**Mean NRSi Reduction (SD)**	**8.5 (1.29)**			

* Discontinued treatment after 4 week follow-up visit due to continued pruritus relief.

## References

[1] Ikoma A., Steinhoff M., Ständer S., Yosipovitch G., Schmelz M. (2006). The neurobiology of itch. Nat. Rev. Neurosci..

[2] Shive M., Linos E., Berger T., Wehner M., Chren M.M. (2013). Itch as a patient-reported symptom in ambulatory care visits in the United States. J. Am. Acad. Dermatol..

[3] Mollanazar N.K., Koch S.D., Yosipovitch G. (2015). Epidemiology of Chronic Pruritus: Where Have We Been and Where Are We Going?. Curr. Dermatol. Rep..

[4] Matterne U., Apfelbacher C.J., Loerbroks A., Schwarzer T., Büttner M., Ofenloch R., Diepgen T.L., Weisshaar E. (2011). Prevalence, correlates and characteristics of chronic pruritus: A population-based cross-sectional study. Acta Derm. Venereol..

[5] Ständer S., Schäfer I., Phan N.Q., Blome C., Herberger K., Heigel H., Augustin M. (2010). Prevalence of chronic pruritus in Germany: Results of a cross-sectional study in a sample working population of 11,730. Dermatology.

[6] Wolkenstein P., Grob J.J., Bastuji-Garin S., Ruszczynski S., Roujeau J.-C., Revuz J. (2003). French people and skin diseases: Results of a survey using a representative sample. Arch. Dermatol..

[7] Ständer S., Weisshaar E., Mettang T., Szepietowski J.C., Carstens E., Ikoma A., Bergasa N., Gieler U., Misery L., Wallengren J. (2007). Clinical classification of itch: A position paper of the International Forum for the Study of Itch. Acta Derm. Venereol..

[8] Papoiu A.D., Coghill R.C., Kraft R.A., Wang H., Yosipovitch G. (2012). A tale of two itches. Common features and notable differences in brain activation evoked by cowhage and histamine induced itch. Neuroimage.

[9] Papoiu A.D.P., Kraft R.A., Coghill R.C., Yosipovitch G. (2015). Butorphanol suppression of histamine itch is mediated by nucleus accumbens and septal nuclei: A pharmacological fMRI study. J. Investig. Dermatol..

[10] Mochizuki H., Papoiu A.D.P., Yosipovitch G., Carstens E.A.T. (2014). Chapter 23 Brain Processing of Itch and Scratching. Itch: Mechanisms and Treatment.

[11] Kini S.P., DeLong L.K., Veledar E., McKenzie-Brown A.M., Schaufele M., Chen S.C. (2011). The impact of pruritus on quality of life: The skin equivalent of pain. Arch. Dermatol..

[12] Zachariae R., Zachariae C., Ibsen H.H., Mortensen J.T., Wulf H.C. (2004). Psychological symptoms and quality of life of dermatology outpatients and hospitalized dermatology patients. Acta Derm. Venereol..

[13] Yosipovitch G., Goon A., Wee J., Chan Y.H., Goh C.L. (2000). The prevalence and clinical characteristics of pruritus among patients with extensive psoriasis. Br. J. Dermatol..

[14] Zachariae R., Zachariae C.O., Lei U., Pedersen A.F. (2008). Affective and sensory dimensions of pruritus severity: Associations with psychological symptoms and quality of life in psoriasis patients. Acta Derm. Venereol..

[15] Mollanazar N.K., Smith P.K., Yosipovitch G. (2016). Mediators of Chronic Pruritus in Atopic Dermatitis: Getting the Itch Out?. Clin. Rev. Allergy Immunol..

[16] Oetjen L.K., Mack M.R., Feng J., Whelan T.M., Niu H., Guo C.J., Chen S., Trier A.M., Xu A.J., Tripathi S.V. (2017). Sensory Neurons Co-opt Classical Immune Signaling Pathways to Mediate Chronic Itch. Cell.

[17] Mollanazar N.K., Sethi M., Rodriguez R.V., Nattkemper L.A., Ramsey F.V., Zhao H., Yosipovitch G. (2016). Retrospective analysis of data from an itch center: Integrating validated tools in the electronic health record. J. Am. Acad. Dermatol..

[18] Mollanazar N.K., Elgash M., Weaver L., Valdes-Rodriguez R., Hsu S. (2018). Reduced itch associated with dupilumab treatment in 4 patients with prurigo nodularis. JAMA Dermatol..

[19] Trier A.M., Kim B.S. (2018). Cytokine modulation of atopic itch. Curr. Opin. Immunol..

[20] Yosipovitch G., Ständer S., Kerby M.B., Larrick J.W., Perlman A.J., Schnipper E.F., Zhang X., Tang J.Y., Luger T., Steinhoff M. (2018). Serlopitant for the treatment of chronic pruritus: Results of a randomized, multicenter, placebo-controlled phase 2 clinical trial. J. Am. Acad. Dermatol..

[21] Iking A., Grundmann S., Chatzigeorgakidis E., Phan N.Q., Klein D., Ständer S. (2013). Prurigo as a symptom of atopic and non-atopic diseases: Aetiological survey in a consecutive cohort of 108 patients. J. Eur. Acad. Dermatol. Venereol..

[22] Boozalis E., Tang O., Patel S., Semenov Y.R., Pereira M.P., Stander S., Kang S., Kwatra S.G. (2018). Ethnic differences and comorbidities of 909 prurigo nodularis patients. J. Am. Acad. Dermatol..

[23] Johansson O., Liang Y., Emtestam L. (2002). Increased nerve growth factor- and tyrosine kinase A-like immunoreactivities in prurigo nodularis skin—An exploration of the cause of neurohyperplasia. Arch. Dermatol. Res..

[24] Groneberg D.A., Serowka F., Peckenschneider N., Artuc M., Grützkau A., Fischer A., Henz B.M., Welker P. (2005). Gene expression and regulation of nerve growth factor in atopic dermatitis mast cells and the human mast cell line-1. J. Neuroimmunol..

[25] Sonkoly E., Muller A., Lauerma A.I., Pivarsi A., Soto H., Kemeny L., Alenius H., Dieu-Nosiean M.C., Meller S., Rieker J. (2006). IL-31: A new link between T cells and pruritus in atopic skin inflammation. J. Allergy Clin. Immunol..

[26] Weigelt N., Metze D., Ständer S. (2010). Prurigo nodularis: Systematic analysis of 58 histological criteria in 136 patients. J. Cutan Pathol..

[27] Schuhknecht B., Marziniak M., Wissel A., Phan N.Q., Pappai D., Dangelmaier J., Ständer S. (2011). Reduced intraepidermal nerve fibre density in lesional and nonlesional prurigo nodularis skin as a potential sign of subclinical cutaneous neuropathy. Br. J. Dermatol..

[28] Calugareanu A., Jachiet M., Lepelletier C., Masson A.D., Rybojad M., Bagot M., Bouaziz J.D. (2019). Dramatic improvement of generalized prurigo nodularis with dupilumab. J. Eur. Acad. Dermatol. Venereol..

[29] Beck K.M., Yang E.J., Sekhon S., Bhutani T., Liao W. (2019). Dupilumab Treatment for Generalized Prurigo Nodularis. JAMA Dermatol..

[30] Fukushi S., Yamasaki K., Aiba S. (2011). Nuclear localization of activated STAT6 and STAT3 in epidermis of prurigo nodularis. Br. J. Dermatol..

[31] Haas S., Capellino S., Phan N.Q., Böhm M., Luger T.A., Straub R.H., Ständer S. (2010). Low density of sympathetic nerve fibers relative to substance P-positive nerve fibers in lesional skin of chronic pruritus and prurigo nodularis. J. Dermatol. Sci..

[32] Steinhoff M.S., von Mentzer B., Geppetti P., Pothoulakis C., Bunnett N.W. (2014). Tachykinins and their receptors: Contributions to physiological control and the mechanisms of disease. Physiol. Rev..

[33] Church M.K., Okayama Y., el-Lati S. (1991). Mediator secretion from human skin mast cells provoked by immunological and non-immunological stimulation. Skin. Pharmacol..

[34] Frenkl T.L., Zhu H., Reiss T., Seltzer O., Rosenberg E., Green S. (2010). A multicenter, double-blind, randomized, placebo controlled trial of a neurokinin-1 receptor antagonist for overactive bladder. J. Urol..

[35] Ständer S., Kwon P., Hirman J., Perlman A.J., Weisshaar E., Metz M., Luger T.A., TCP-102 Study Group (2019). Serlopitant reduced pruritus in patients with prurigo nodularis in a phase 2, randomized, placebo-controlled trial. J. Am. Acad. Dermatol..

[36] Matsuda K.M., Sharma D., Schonfeld A.R., Kwatra S.G. (2016). Gabapentin and pregabalin for the treatment of chronic pruritus. J. Am. Acad. Dermatol..

[37] Mazza M., Guerriero G., Marano G., Janiri L., Bria P., Mazza S. (2013). Treatment of prurigo nodularis with pregabalin. J. Clin. Pharm. Ther..

[38] Saraceno R., Chiricozzi A., Nisticò S.P., Tiberti S., Chimenti S. (2010). An occlusive dressing containing betamethasone valerate 0.1% for the treatment of prurigo nodularis. J. Dermatolog. Treat..

[39] Siepmann D., Lotts T., Blome C., Braeutigam M., Phan N.Q., Butterfass-Bahloul T., Augustin M., Luger T.A., Ständer S. (2013). Evaluation of the antipruritic effects of topical pimecrolimus in non-atopic prurigo nodularis: Results of a randomized, hydrocortisone-controlled, double-blind phase II trial. Dermatology.

[40] Andersen T.P., Fogh K. (2011). Thalidomide in 42 patients with prurigo nodularis Hyde. Dermatology.

[41] Siepmann D., Luger T.A., Ständer S. (2008). Antipruritic effect of cyclosporine microemulsion in prurigo nodularis: Results of a case series. J. Dtsch. Dermatol. Ges..

[42] Aguh C., Kwatra S.G., He A., Okoye G.A. (2018). Thalidomide for the treatment of chronic refractory prurigo nodularis. Dermatol. Online J..

[43] Valdes-Rodriguez R., Stull C., Yosipovitch G. (2015). Chronic pruritus in the elderly: Pathophysiology, diagnosis and management. Drugs Aging.

[44] Xu A.Z., Tripathi S.V., Kau A.L., Schaffer A., Kim B.S. (2016). Immune dysregulation underlies a subset of patients with chronic pruritus of unknown origin. J. Am. Acad. Dermatol..

[45] Wills-Karp M., Finkelman F.D. (2008). Untangling the complex web of IL-4- and IL-13-mediated signaling pathways. Sci. Signal..

[46] Shevchenko A., Valdes-Rodriguez R., Yosipovitch G. (2018). Causes, pathophysiology, and treatment of pruritus in the mature patient. Clin. Dermatol..

[47] Maurer M., Rosén K., Hsieh H.J., Saini S., Grattan C., Gimenéz-Arnau A., Agarwal S., Doyle R., Canvin J., Kaplan A. (2013). Omalizumab for the treatment of chronic idiopathic or spontaneous urticaria. N. Engl. J. Med..

[48] Wenzel S., Ford L., Pearlman D., Spector S., Sher L., Skobieranda F., Wang L., Kirkesseli S., Rocklin R., Bock B. (2013). Dupilumab in persistent asthma with elevated eosinophil levels. N. Engl. J. Med..

[49] Wenzel S., Castro M., Corren J., Maspero J., Wang L., Zhang B., Pirozzi G., Sutherland E.R., Evans R.R., Joish V.N. (2016). Dupilumab efficacy and safety in adults with uncontrolled persistent asthma despite use of medium-to-high-dose inhaled corticosteroids plus a long-acting β2 agonist: A randomised double-blind placebo-controlled pivotal phase 2b dose-ranging trial. Lancet.

[50] Blauvelt A., de Bruin-Weller M., Gooderham M., Cather J.C., Weisman J., Pariser D., Simpson E.L., Papp K.A., Hong H.C.H., Rubel D. (2017). Long-term management of moderate-to-severe atopic dermatitis with dupilumab and concomitant topical corticosteroids (LIBERTY AD CHRONOS): A 1-year, randomised, double-blinded, placebo-controlled, phase 3 trial. Lancet.

[51] Bihari B. (1995). Efficacy of low dose naltrexone as an immune stabilizing agent for the treatment of HIV/AIDS. AIDS Patient Care.

[52] Ekelem C., Juhasz M., Khera P., Mesinkovska N.A. (2019). Utility of Naltrexone Treatment for Chronic Inflammatory Dermatologic Conditions: A Systematic Review. JAMA Dermatol..

[53] Metze D., Reimann S., Beissert S., Luger T. (1999). Efficacy and safety of naltrexone, an oral opiate receptor antagonist, in the treatment of pruritus in internal and dermatological diseases. J. Am. Acad. Dermatol..

[54] Weston G., Payette M. (2015). Update on lichen planus and its clinical variants. Int. J. Womens Dermatol..

[55] Usatine R.P., Tinitigan M. (2011). Diagnosis and treatment of lichen planus. Am. Fam. Physician.

[56] Reich A., Welz-Kubiak K., Szepietowski J.C. (2011). Pruritus differences between psoriasis and lichen planus. Acta Derm. Venereol..

[57] Welz-Kubiak K., Reich A. (2013). Mediators of pruritus in lichen planus. Autoimmune Dis..

[58] Yin M., Li G., Song H., Lin S. (2017). Identifying the association between interleukin-6 and lichen planus: A meta-analysis. Biomed. Rep..

[59] Sun A., Chia J.S., Chang Y.F., Chiang C.P. (2002). Serum interleukin-6 level is a useful marker in evaluating therapeutic effects of levamisole and Chinese medicinal herbs on patients with oral lichen planus. J. Oral. Pathol. Med..

[60] Rhodus N.L., Cheng B., Bowles W., Myers S., Miller L., Ondrey F. (2006). Proinflammatory cytokine levels in saliva before and after treatment of (erosive) oral lichen planus with dexamethasone. Oral Dis..

[61] Diehl S., Rincón M. (2002). The two faces of IL-6 on Th1/Th2 differentiation. Mol. Immunol..

[62] Sukul N., Speyer E., Tu C., Bieber B.A., Li Y., Lopes A.A., Asahi K., Mariani L., Laville M., Rayner H.C. (2019). Pruritus and Patient Reported Outcomes in Non-Dialysis CKD. Clin. J. Am. Soc. Nephrol..

[63] Pisoni R.L., Wikström B., Elder S.J., Akizawa T., Asano Y., Keen N.L., Saran R., Mendelssohn D.C., Young E.W., Port F.K. (2006). Pruritus in haemodialysis patients: International results from the Dialysis Outcomes and Practice Patterns Study (DOPPS). Nephrol. Dial. Transplant..

[64] Gangemi S., Quartuccio S., Casciaro M., Trapani G., Minciullo P.L., Imbalzano E. (2017). Interleukin 31 and skin diseases: A systematic review. Allergy Asthma Proc..

[65] Simonsen E., Komenda P., Lerner B., Askin N., Bohm C., Shaw J., Tangri N., Rigatto C. (2017). Treatment of Uremic Pruritus: A Systematic Review. Am. J. Kidney Dis..

[66] Taylor C.P., Gee N.S., Su T.Z., Kocsis J.D., Welty D.F., Brown J.P., Dooley D., Boden P., Singh L. (1998). A summary of mechanistic hypotheses of gabapentin pharmacology. Epilepsy Res..

[67] Li Z., Taylor C.P., Weber M., Piechan J., Prior F., Bian F., Cui M., Hoffman D., Donevan S. (2011). Pregabalin is a potent and selective ligand for α(2)δ-1 and α(2)δ-2 calcium channel subunits. Eur. J. Pharmacol..

[68] Quintero J.E., Dooley D.J., Pomerleau F., Huettl P., Gerhardt G.A. (2011). Amperometric measurement of glutamate release modulation by gabapentin and pregabalin in rat neocortical slices: Role of voltage-sensitive Ca2+ α2δ-1 subunit. J. Pharmacol. Exp. Ther..

[69] Gunal A.I., Ozalp G., Yoldas T.K., Gunal S.Y., Kirciman E., Celiker H. (2004). Gabapentin therapy for pruritus in haemodialysis patients: A randomized, placebo-controlled, double-blind trial. Nephrol. Dial. Transplant..

[70] Naini A.E., Harandi A.A., Khanbabapour S., Shahidi S., Seirafiyan S., Mohseni M. (2007). Gabapentin: A promising drug for the treatment of uremic pruritus. Saudi J. Kidney Dis. Transplant..

[71] Silverberg J.I., Brieva J. (2019). A successful case of dupilumab treatment for severe uremic pruritus. JAAD Case Rep..

[72] Barzilai A., Shpiro D., Goldberg I., Yacob-Hirsch Y., Diaz-Cascajo C., Meytes D., Schiby R., Amariglio N., Trau H. (1999). Insect bite-like reaction in patients with hematologic malignant neoplasms. Arch. Dermatol..

[73] Byrd J.A., Scherschun L., Chaffins M.L., Fivenson D.P. (2001). Eosinophilic dermatosis of myeloproliferative disease: Characterization of a unique eruption in patients with hematologic disorders. Arch. Dermatol..

[74] Davis M.D., McEvoy M.T. (2002). Eosinophilic dermatosis associated with hematologic disorders: Not so unique. Arch. Dermatol..

[75] Davis M.D., Perniciaro C., Dahl P.R., Randle H.W., McEvoy M.T., Leiferman K.M. (1998). Exaggerated arthropod-bite lesions in patients with chronic lymphocytic leukemia: A clinical, histopathologic, and immunopathologic study of eight patients. J. Am. Acad. Dermatol..

[76] Farber M.J., La Forgia S., Sahu J., Lee J.B. (2012). Eosinophilic dermatosis of hematologic malignancy. J. Cutan. Pathol..

[77] Qiao J., Sun C.E., Zhu W., Zhu D., Fang H. (2013). Flame figures associated with eosinophilic dermatosis of hematologic malignancy: Is it possible to distinguish the condition from eosinophilic cellulitis in patients with hematoproliferative disease?. Int. J. Clin. Exp. Pathol..

[78] Bari O., Cohen P.R. (2017). Eosinophilic dermatosis of hematologic malignancy mimicking varicella zoster infection: Report in a woman with chronic lymphocytic leukemia and review of the literature. Dermatol. Pract. Concept..

[79] Penn L., Ahern I., Mir A., Meehan S.A. (2015). Eosinophilic dermatitis of hematologic malignancy. Dermatol. Online J..

[80] Bairey O., Goldschmidt N., Ruchlemer R., Tadmor T., Rahimi-Levene N., Yuklea M., Shvidel L., Berrebi A., Polliack A., Herishanu Y. (2012). Insect-bite-like reaction in patients with chronic lymphocytic leukemia: A study from the Israeli Chronic Lymphocytic Leukemia Study Group. Eur. J. Haematol..

[81] Jayasekera P.S., Bakshi A., Al-Sharqi A. (2016). Eosinophilic dermatosis of haematological malignancy. Clin. Exp. Dermatol..

[82] Meiss F., Technau-Hafsi K., Kern J.S., May A.M. (2019). Eosinophilic dermatosis of hematologic malignancy: Correlation of molecular characteristics of skin lesions and extracutaneous manifestations of hematologic malignancy. J. Cutan. Pathol..

